# Chitosan-Based Accelerated Portland Cement Promotes Dentinogenic/Osteogenic Differentiation and Mineralization Activity of SHED

**DOI:** 10.3390/polym13193358

**Published:** 2021-09-30

**Authors:** Hasan Subhi, Adam Husein, Dasmawati Mohamad, Nik Rozainah Nik Abdul Ghani, Asma-Abdullah Nurul

**Affiliations:** 1 School of Dental Sciences, Universiti Sains Malaysia, Kubang Kerian 16150, KTN, Malaysia; hsnsuaz@gmail.com (H.S.); dasmawati@usm.my (D.M.); nikroza@gmail.com (N.R.N.A.G.); 2 School of Health Sciences, Universiti Sains Malaysia, Kubang Kerian 16150, KTN, Malaysia

**Keywords:** chitosan, cytotoxicity, dental tissue engineering, dentinogenic/osteogenic differentiation, endodontic, Portland cement

## Abstract

Calcium silicate-based cements (CSCs) are widely used in various endodontic treatments to promote wound healing and hard tissue formation. Chitosan-based accelerated Portland cement (APC-CT) is a promising and affordable material for endodontic use. This study investigated the effect of APC-CT on apoptosis, cell attachment, dentinogenic/osteogenic differentiation and mineralization activity of stem cells from human exfoliated deciduous teeth (SHED). APC-CT was prepared with various concentrations of chitosan (CT) solution (0%, 0.625%, 1.25% and 2.5% (*w*/*v*)). Cell attachment was determined by direct contact analysis using field emission scanning electron microscopy (FESEM); while the material extracts were used for the analyses of apoptosis by flow cytometry, dentinogenic/osteogenic marker expression by real-time PCR and mineralization activity by Alizarin Red and Von Kossa staining. The cells effectively attached to the surfaces of APC and APC-CT, acquiring flattened elongated and rounded-shape morphology. Treatment of SHED with APC and APC-CT extracts showed no apoptotic effect. APC-CT induced upregulation of *DSPP, MEPE, DMP-1, OPN, OCN, OPG* and *RANKL* expression levels in SHED after 14 days, whereas *RUNX2, ALP* and *COL1A1* expression levels were downregulated. Mineralization assays showed a progressive increase in the formation of calcium deposits in cells with material containing higher CT concentration and with incubation time. In conclusion, APC-CT is nontoxic and promotes dentinogenic/osteogenic differentiation and mineralization activity of SHED, indicating its regenerative potential as a promising substitute for the commercially available CSCs to induce dentin/bone regeneration.

## 1. Introduction

Calcium silicate-based cements (CSCs) have been widely used in different endodontic treatments such as vital pulp therapy (pulp capping and pulpotomy), apexification, apicoectomy and repair of perforation and resorption [1,2]. In endodontic treatments, bioactive materials are applied in direct contact with the vital pulp and periradicular tissue, which requires the materials to be biocompatible, induce healing and hard tissue formation and be hydrophilic to set in a moist environment [3,4]. CSCs materials that have been synthesized for dental applications are mainly based on dicalcium and tricalcium silicates such as ProRoot mineral trioxide aggregate (MTA), BioAggregate and Biodentine [2]. These materials were reported to possess superior biocompatibility properties and the ability to induce hard tissue formation [5,6]. However, the long setting time, high cost, tooth discoloration and difficult handling properties are the main drawbacks of ProRoot MTA [7]. Although BioAggregate exhibited comparable favorable properties compared to MTA, its poorer mechanical characteristics and long setting time limit its ability to replace MTA [8]. Furthermore, Biodentine was reported to have low radiopacity [9]. Portland cement (PC) is a calcium silicate-based cement. It is a cheaper and widely available material with similar chemical composition (except for bismuth) and biological properties to MTA [10,11,12,13], which encouraged researchers to study and modify PC for use in dentistry as an endodontic material [14,15,16]. However, PC has a long setting time and requires modification to the material for more efficient use in the clinic. Thus, calcium chloride was added to accelerate the setting time [17,18], and this material was called accelerated PC (APC). APC exhibits favorable physicochemical properties [18], biocompatibility and the ability to induce hard tissue repair [10,19]. Hence, the material addresses some drawbacks of conventional materials and has been recommended to be used as an affordable and low-cost material in dental applications. The ability of a material to induce the formation of hard tissue is essential for tissue regeneration. As a result, adding an additive to enhance the APC’s potential for dentin and bone regeneration, as well as its biocompatibility and physical and mechanical properties, will improve the material’s qualities and accelerate tissue regeneration. Chitosan (CT) is a natural cationic polymer with outstanding biological properties such as biocompatibility, low allergenicity, nontoxicity, biodegradability, antimicrobial, hemostatic, antitumor and antioxidant [20] making it a versatile material in tissue engineering to regenerate various tissues such as dentin, bone, skin, nerves, cartilage and muscle [21,22]. It is effective in producing biomimetically mineralized composite materials with good bionic properties such as dentinogenic/osteogenic potential, mechanical properties, biocompatibility and bioactivity. Thus, CT is interesting for its potential to be applied alone or in combination with other materials for various aspects of bone and dental tissue engineering [22]. Assessment of the biocompatibility of endodontic materials is essential to avoid the cytotoxic effect from degradation products or elution substances on pulpal and periradicular cells which may trigger cell death by apoptosis or necrosis [23]. In addition, for a successful endodontic treatment, the material used must be neutral or stimulate repair to promote tooth healing and function [24]. The mechanism of the biological activity of the material on dental stem cells has a key role in tissue repair. Cell culture is a widely used effective model system to study the cellular behavior and biological response to specific conditions including response to material properties such as cytotoxicity and cell differentiation potential [25] before the in vivo studies. Stem cells from human exfoliated deciduous teeth (SHED) are dental tissue-derived mesenchymal stem cells (MSCs). They have been identified as a population of high proliferative capacity and potential for differentiation into various cell types such as neural cells, adipocytes, odontoblasts and osteoblasts, and they contribute to dentin and bone formation in vivo [26,27]. In our recent study, a new chitosan-based APC (APC-CT) was synthesized and characterized [28]. The new material was composed of APC and CT and designed for endodontic applications. APC-CT demonstrated appropriate physicochemical and mechanical properties, was able to support cell proliferation and exhibited favorable interaction with the cells, indicating that the new material is a promising and affordable alternative to conventional endodontic materials. In the present study, the cytotoxicity, dentinogenic/osteogenic differentiation potential and mineralization activity of APC-CT were evaluated on stem cells isolated from the human deciduous teeth (SHED).

## 2. Materials and Methods

### 2.1. Preparation of APC-CT

APC-CT was synthesized as described previously [28]. Briefly, white Portland cement powder (PC; Aalborg, Perak, Malaysia) was sieved (63 µm sieve; Retsch, Haan, Germany, ISO 3310-1) to obtain a homogeneous powder as described earlier [19,28] and mixed with 10% (*w*/*w*) calcium chloride dihydrate (CaCl_2_ × 2H_2_O) (Merck, Darmstadt, Germany) to form the powder component. To prepare CT solutions, CT powder (a practical grade, Sigma-Aldrich, Reykjavik, Iceland; ≥75% degree of deacetylation; molecular weight of 190–375 KDa) was dissolved in 1% (*v*/*v*) acetic acid solution (Merck, Darmstadt, Germany) using magnetic stirring for 3 h. Three different concentrations of CT solutions of 2.5%, 1.25% and 0.625% (*w*/*v*) were prepared by dissolving 2.5, 1.25 and 0.625 g of CT powder in 100 mL of 1% acetic acid solution, respectively, and then filtered by passing through 0.45 µm filter. APC mixed with distilled water was used as control.

Then, the APC powder component was mixed with liquid component (CT solutions in APC-CT or distilled water in APC) using the optimal liquid to powder ratios that obtained the best material consistency and handling properties, whereby 1 g of PC and 0.1 g of CaCl_2._2H_2_O were mixed with 0.250, 0.280, 0.300 and 0.320 mL of liquid component to prepare APC, APC-0.6%CT, APC-1.25%CT and APC-2.5%CT, respectively. The test materials were placed into acrylic molds (5 mm in diameter and 2 mm in height) and allowed to set as described previously [28]. The samples were then sterilized by ultraviolet light for 30 min (15 min for each side) as described previously [29].

### 2.2. Preparation of the Material Extracts

The material discs were incubated in complete culture medium prepared from alpha minimum essential medium (α-MEM) supplemented with 10% (*v*/*v*) FBS and 1% (*v*/*v*) penicillin–streptomycin solution (all from Gibco, Grand Island, NY, USA) for 3 days in 37 °C, 5% CO_2_ and 100% humidity. The culture medium was supplemented with dentinogenic/osteogenic induction reagents (OM) consisting of 50 µg/mL L-ascorbic acid, 10 nM dexamethasone and 10 mM β-glycerophosphate (all from Sigma-Aldrich, St. Louis, MO, USA) in apoptosis, dentinogenic/osteogenic differentiation and mineralization assays. The material extracts at a concentration of 12.5 mg/mL were selected based on our previous work [28] which demonstrated that the highest extract concentration displayed comparable or increased cell viability compared to the control at day 3. After 3 days, the extracts were collected and filtered by passing through a sterile 0.22 µm filter. The material-free culture medium supplemented with OM, incubated under identical conditions, served as control. 

### 2.3. Cell Culture

**Figure 1:**
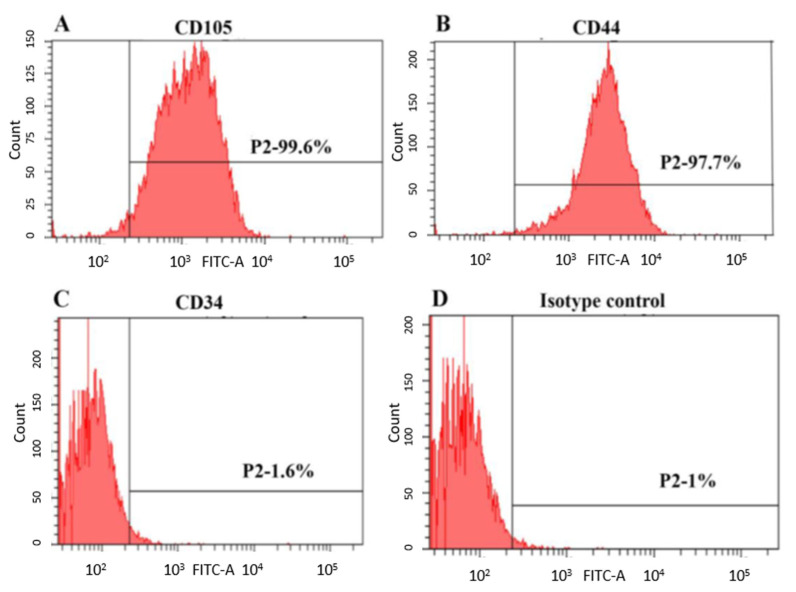
Immunophenotypic characterization of SHED analyzed by flow cytometry. Representative diagram showing the expression of cell surface markers (**A**) CD105, (**B**) CD44, (**C**) CD34 and (**D**) isotype control.

SHED employed in this study were obtained from ALLCELLS, USA (Catalog No. DP004F; lot number SHED092211-01). Upon being received, the cells were characterized using stem cell markers CD105, CD44 and CD34 by flow cytometry (Figure 1). The cells were cultured in complete culture medium consisting of α-MEM supplemented with 10% FBS and 1% penicillin–streptomycin solution (all from Gibco, Grand Island, NY, USA) and incubated at 37 °C in 5% CO_2_ and 95% humidity. The culture medium was changed every 3 days; SHED from passages 5–7 were used in this study.

### 2.4. Apoptosis Assay

The material’s effects on cell death and apoptosis were investigated by FITC Annexin V Apoptosis Detection kit 1 (BD Biosciences, San Jose, CA, USA). Briefly, SHED in a density of 5.0 × 10^4^ were cultured in 6-well plates for 24 h in a humidified incubator at 37 °C in 5% CO_2_. The cells were then treated with the material extract with or without OM and incubated in a humidified incubator in 5% CO_2_ at 37 °C. After 1, 2 and 3 days of incubation, the cells were collected, washed in phosphate buffer saline (PBS) (Gibco, Grand Island, NY, USA) and stained with annexin V-FITC and propidium iodide (PI) according to the manufacturer’s instructions. The cells were analyzed by flow cytometer (FACS Canto II, BD Biosciences, San Jose, CA, USA) and BD FACS Diva software (BD Biosciences, San Jose, CA, USA). Ten thousand events were analyzed for each sample, and each sample was prepared in three biological replicates. 

### 2.5. Assessment of Cell Attachment

To evaluate the cell attachment and viability of the material, SHED-seeded APC and APC-CT were observed after the direct contact by field emission scanning electron microscopy (FESEM) as described earlier [29]. The materials were placed in 6-well plates and seeded with 1.5 × 10^5^ cells on top of the material discs and left for 30 min. The wells were filled with culture medium to cover the seeded cells and then incubated at 37 °C in 5% CO_2_. After 3 days, the cells were washed with distilled water and fixed with 2.5% glutaraldehyde at 4 °C for 2 h and then dehydrated in graded series of ethanol concentrations (30%, 50% for 10 min each; 70%, 90%, 100%, 100% for 5 min each) and dried in a desiccator. Eight material discs (two per group) were prepared for cell attachment examination. Then, the material specimens were gold-coated by a sputter coating machine (EM SCD005, Leica, Wetzlar, Germany) and viewed by FESEM (Quanta FEG 450, Fei, Hillsboro, OR, USA). 

### 2.6. Quantitative Real-Time Polymerase Chain Reaction (qRT-PCR) Analysis

qRT-PCR was performed to detect the relative expression level of the dentinogenic/osteogenic gene markers, i.e., *DSPP, MEPE, DMP-1, OPN, OCN, OPG, RANKL, RUNX2, ALP* and *COL1A1*, in SHED treated with material extracts or material-free culture medium supplemented with OM (as control) for 3, 7 and 14 days. 

Briefly, the total RNA was extracted using innuPREP RNA Mini Kit (Analytikjena, Jena, Germany) according to the manufacturer’s protocol and quantified with a spectrophotometer (Biophotometer plus, Eppendorf, Hamburg, Germany). Melting curve analysis was performed for all genes to ensure their specificity followed by standard curve to select the best template concentration for the PCR amplification. qRT-PCR reaction was performed using ABI Step One Plus PCR system (Applied Biosystems, Foster City, CA, USA) with 50 ng of RNA quantified with SensiFast SYBR Hi-ROX One-Step Kit (Bioline, London, UK) according to the manufacturer’s instructions and supplemented with 200 nM of specific primer set (Table 1). 

qRT-PCR reaction was performed using cycling conditions with reverse transcription at 45 °C for 10 min followed by polymerase activation at 95 °C for 2 min, then 40 cycles of (i) denaturation at 95 °C for 5 s, (ii) annealing at 52.4–60.4 °C for 10 s and (iii) extension at 72 °C for 5 s. The annealing temperature depended on the primer set used. The primer sequences and annealing temperatures of individual genes used in qRT-PCR are listed in Table 1. Each reaction was run in triplicate, and the qRT-PCR was conducted in 3 biological replicates. Nontemplate control (NTC) was included in each assay. The cycle threshold values were obtained, and the relative mRNA levels were normalized to the geometric mean of *β-actin* and *GAPDH* housekeeping genes and calculated using the 2^-ΔΔCt^ method [30]. 

### 2.7. Extracellular Matrix (ECM) Mineralization

The effect of APC and APC-CT on the ECM mineralization activity was assessed by Alizarin Red S and Von Kossa stainings for 14 and 21 days. Totals of 2.0 × 10^4^ and 5.0 × 10^3^ cells/well were cultured in 6-well plates for 14 and 21 days, respectively. After 24 h of seeding, the cells were incubated with the APC and APC-CT extracts. Untreated SHED served as control. 

For Alizarin Red staining, the cells were washed with PBS and fixed in 10% formalin (Sigma-Aldrich, St. Louis, MO, USA) for 45 min at room temperature. Then, the cells were washed with distilled water and stained with 2% Alizarin Red staining (Sigma-Aldrich, St. Louis, MO, USA) solution for 45 min at room temperature, followed by washing with distilled water to stop the reaction. The cell monolayer was then visualized under the inverted microscope (Zeiss, Oberkochen, Germany) and analyzed by Image-Pro Express software (Media Cybernetics, MD, USA). Quantification of the mineralized tissues was conducted using ImageJ software (ImageJ 1.52a, National Institutes of Health, Bethesda, MD, USA).

Von Kossa staining was performed using Silver Staining Kit ACC (Merck, Darmstadt, Germany) following the manufacturer’s instructions. Briefly, SHED were washed with PBS and fixed in 70% ethanol (HmbG, Hamburg, Germany) for 30 min at room temperature. Then, the cells were washed with distilled water, stained with 5% silver nitrate for 30 min in the dark and washed again with distilled water. After that, the cells were exposed to bright light for 25 min and stained with sodium thiosulphate for 5 min. After washing, the cells were stained with nuclear fast red and washed with distilled water and PBS to stop the reaction. The cell monolayer was then visualized under the inverted microscope (Zeiss, Oberkochen, Germany) and analyzed by Image-Pro Express software (Media Cybernetics, MD, USA). Mineralized tissue was quantified using ImageJ software (ImageJ 1.52a, National Institutes of Health, Bethesda, MD, USA). 

### 2.8. Statistical Analysis

Statistical analyses were performed using Statistical Package of Social Science SPSS software (Version 24.0; IBM Corp, Armonk, NY, USA). Data were obtained from three independent experiments. The statistical analyses of apoptosis assay, real-time PCR and Von Kossa staining were carried out by Kruskal–Wallis test and pairwise comparisons. Alizarin Red staining assay was analyzed by one-way analysis of variance (ANOVA) followed by post hoc Dunnett T3 test. Statistical significance was considered at *p* < 0.05 for all tests.

## 3. Results

### 3.1. APC-CT Did Not Exhibit Cytotoxic Effects in SHED

**Figure 2:**
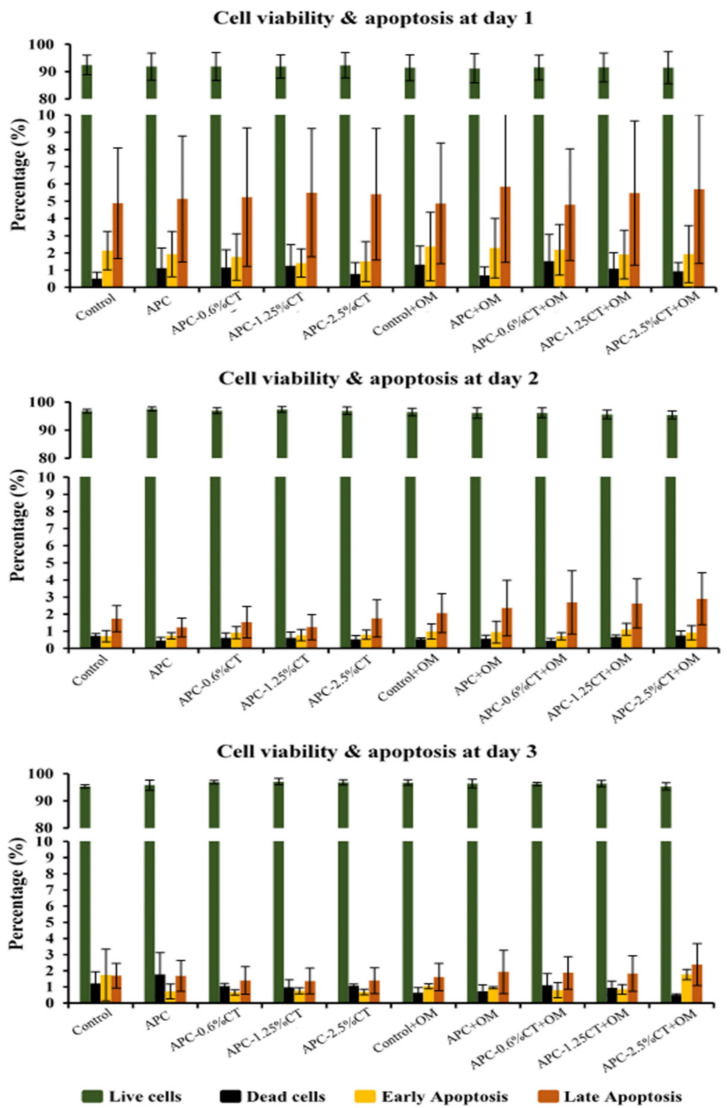
Cytotoxicity induced by APC and APC-CT on SHED using flow cytometry. The graphs present cell population as viable, early apoptotic and late apoptotic cells after 1, 2 and 3 days. Data represent the mean ± SD of three independent experiments (*n* = 3). Statistical analysis indicated no significant difference among the groups at any time point.

Flow cytometry analysis demonstrated that the percentage of live cells increased from >91% on day 1 to >95% on days 2 and 3. Early and late apoptosis dropped from <2.4% and <5.9% on day 1 to <1.8% and <2.4% on day 3, respectively. The results showed that the percentages of live, early apoptotic, and late apoptotic cells treated with the materials with or without OM did not significantly differ (*p* > 0.05) from the control group (untreated SHED) or between the treated groups at any time point. The averages of live and apoptotic cells treated with the test materials are shown in Figure 2.

**Figure 3:**
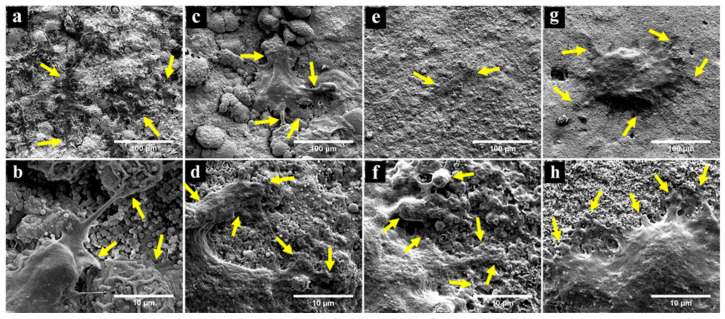
FESEM observation showing successful attachment and spread of SHED on (**a**,**b**) APC, (**c**,**d**) APC-0.6%CT, (**e**,**f**) APC-1.25%CT and (**g**,**h**) APC-2.5%CT after 3 days. Yellow arrows in upper images (**a**,**c**,**e**,**g**) focus on cell attachment and extension of cytoplasmic processes. Yellow arrows in high-magnification images (**b**,**d**,**f**,**h**) point to the interface between lamellipodial and filopodial protrusions and the material surface. Magnification of 1000× (upper) and 10,000× (lower).

FESEM observation of SHED seeded in direct contact with the materials for 3 days demonstrated that the cells spread and proliferated on the surfaces of the materials and maintained a flat elongated morphology with cytoplasmic extensions as shown in Figure 3. The cells on APC, APC-0.6%CT and APC-1.25%CT appeared spindle-like in shape (Figure 3a,c,e). Meanwhile, a flat cell with rounded-shape morphology was observed on APC-2.5%CT (Figure 3g). Lamellipodia and filopodial processes extended from cells to the surrounding materials, which are indicative of effective cell attachment (Figure 3b,d,f,h). 

### 3.2. APC-CT Promoted Dentinogenic/Osteogenic Differentiation in SHED

**Figure 4:**
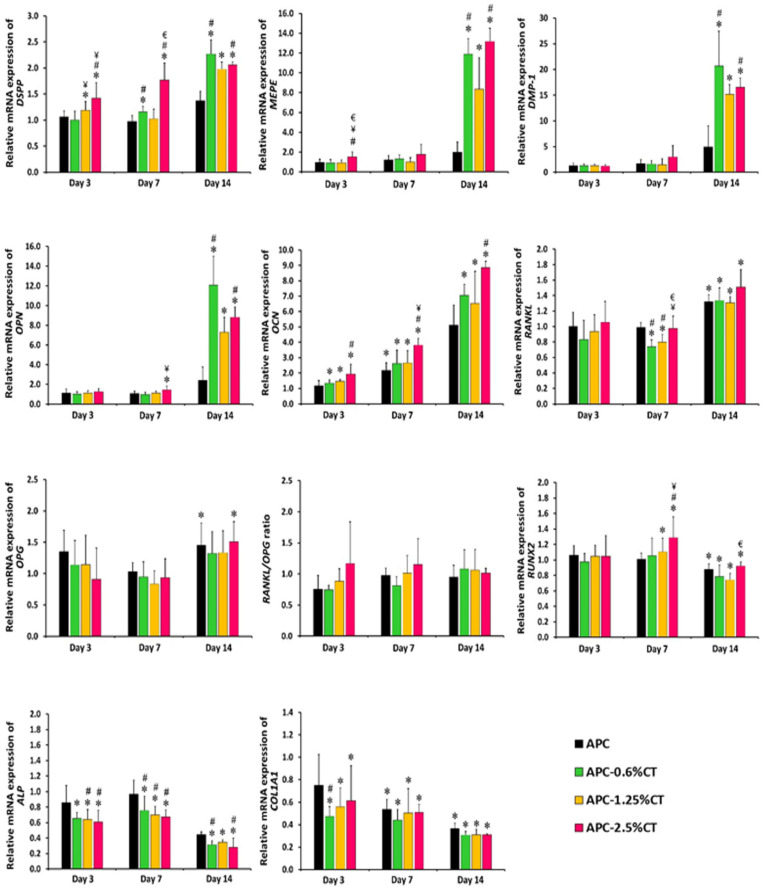
Real-time PCR analysis of relative mRNA expression of dentinogenic/osteogenic-related markers, including DSPP, MEPE, DMP-1, OPN, OCN, OPG, RANKL, RUNX2, ALP and COL1A1 genes in SHED exposed to APC and APC-CT materials after 3, 7 and 14 days. The relative gene expression of target genes was normalized against the internal control genes (GAPDH and β-actin) and relative to untreated control. The control was set at 1 and the data represent mean ± SD of three samples in three independent experiments (*n* = 3). * *p* < 0.05 vs. control, ^#^ *p* < 0.05 vs. APC, ^¥^ *p* < 0.05 vs. APC-0.6%CT and ^€^ *p* < 0.05 vs. APC-1.25%CT.

The effects of experimental endodontic material on the expression levels of dentinogenic and osteogenic markers on days 3, 7 and 14 are presented in Figure 4. *DSPP* expression was upregulated (*p* < 0.05) in cells treated with APC-1.25%CT and APC-2.5%CT relative to control on day 3, while APC-0.6%CT and APC-2.5%CT were upregulated on day 7. *MEPE* and *DMP-1* expression levels were similar (*p* > 0.05) in experimental groups relative to control on days 3 and 7. The secretion of *DSPP*, *MEPE* and *DMP-1* was increased on day 14, showing statistically higher expression levels in CT-containing materials relative to control and in APC-0.6%CT and APC-2.5%CT groups compared to APC.

*OPN* expression was similar (*p* > 0.05) in experimental groups relative to control on days 3 and 7, except for the APC-2.5%CT group on day 7. The expression of *OPN* was then increased on day 14, showing statistically higher expression levels in CT-containing materials relative to control and in APC-0.6%CT and APC-2.5%CT groups compared to APC. *OCN* expression increased (*p* < 0.05) in CT-containing materials relative to control and in APC-2.5%CT groups compared to APC at all time points.

The expression level of *OPG* expression was similar (*p* > 0.05) in the experimental groups relative to control on days 3 and 7 and then increased on day 14, showing a significant difference in APC and APC-2.5%CT groups relative to control. Meanwhile, *RANKL* expression was similar (*p* > 0.05) to control on day 3 and decreased in APC-0.6%CT and APC-1.25%CT groups on day 7. However, it increased on day 14 relative to control. The RANKL/OPG ratio did not demonstrate statistical significance (*p* > 0.05) in the fold difference between the APC-CT-treated groups or relative to control at any treatment durations.

*RUNX2* expression was similar (*p >* 0.05) in experimental groups relative to control on day 3 and increased on day 7, showing a significant difference in APC-1.25%CT and APC-2.5%CT groups. Then, it was downregulated (*p* < 0.05) in all experimental groups on day 14. The expression levels of *ALP* and *COL1A1* were downregulated (*p* < 0.05) in SHED treated with CT-containing material. In addition, APC also induced downregulation of *COL1A1* expression in SHED on days 7 and 14. 

### 3.3. APC-CT Enhanced Mineralization Activity in SHED

**Figure 5:**
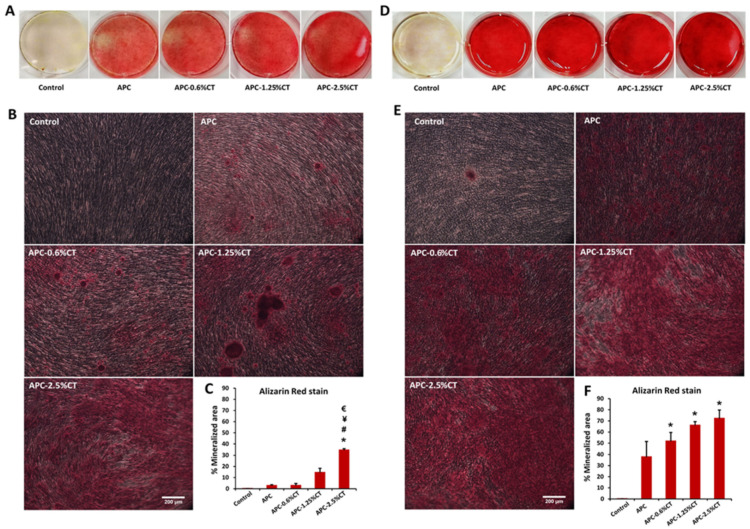
Representative images of calcium deposit formation after (**A**,**B**) 14 and (**D**,**E**) 21 days of SHED exposure to APC and APC-CT as evaluated by Alizarin Red staining. Magnification is 100×. Scale bar = 200 µm. (**C**,**F**) Quantitation of calcium deposition as measured by ImageJ software. The data are presented as mean ± SD after 14 and 21 days. * *p* < 0.05 vs. control, ^#^ *p* < 0.05 vs. APC, ^¥^ *p* < 0.05 vs. APC-0.6%CT and ^€^ *p* < 0.05 vs. APC-1.25%CT, (*n* = 3).

The formation of extracellular matrix by SHED exposed to APC and APC-CT was assessed by Alizarin Red and Von Kossa stainings to evaluate the calcium deposition. In Alizarin Red staining, SHED treated with APC for 14 days exhibited mild mineralized depositions that were increased on day 21 (Figure 5). These depositions were highly distinguishable compared to control (untreated SHED). The mineralization areas in APC-CT groups were significantly higher than APC and showed a gradual increase in area and quantity of red precipitations with material containing higher CT concentration and with incubation time. Untreated SHED demonstrated a low level of calcium deposition on day 21. The quantitative analysis recorded a statistically significant increase in the mineralization in SHED treated with APC-2.5%CT compared to other groups on day 14 (*p* < 0.05), as well as in SHED treated with APC-CT compared to APC and control on day 21 (*p* < 0.05). 

**Figure 6:**
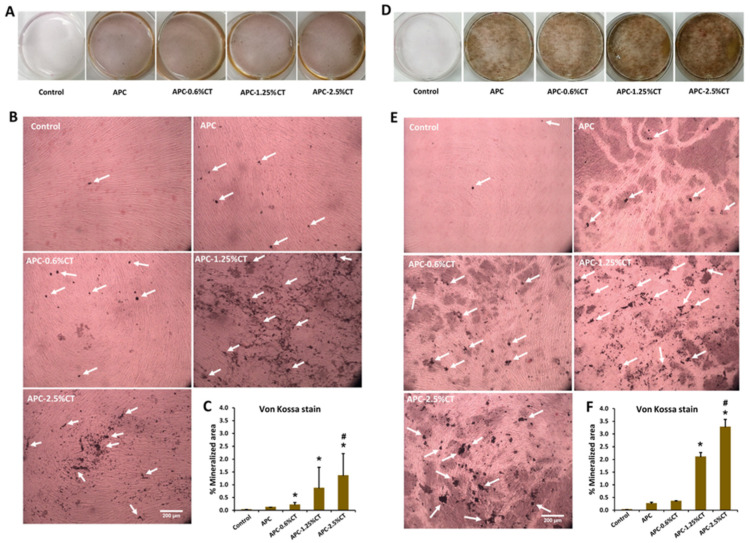
Representative images of mineralized tissue formation after (**A**,**B**) 14 and (**D**,**E**) 21 days of SHED exposure to APC and APC-CT as evaluated by Von Kossa stain. White arrow indicates mineralized matrix. Magnification is 100×. Scale bar = 200 µm. (**C**,**F**) Quantitation of mineral deposition as measured by ImageJ software. The data are presented as mean ± SD after 14 and 21 days. * *p* < 0.05 vs. control and ^#^ *p* < 0.05 vs. APC (*n* = 3).

Von Kossa stain demonstrated the presence of black deposits with different intensities which indicate the mineralization areas in response to APC and APC-CT as shown in Figure 6. Untreated SHED showed minimal mineralization areas. On days 14 and 21, APC-treated cells exhibited more distributed calcium depositions compared to control. Larger areas and quantities of calcium depositions were shown in SHED treated with APC-CT than in APC and control, and gradual increases in area and quantity were observed with higher CT concentrations and incubation time. 

The quantitative analysis recorded a statistically significant increase in the mineralization areas in SHED treated with APC-2.5%CT compared to control and APC (*p* < 0.05) and in APC-1.25%CT compared to control (*p* < 0.05) on days 14 and 21. Moreover, APC-0.6%CT demonstrated statistically higher mineralization areas compared to control on day 14. 

## 4. Discussion

The materials used in endodontics treatments should be biocompatible and exhibit dentinogenic and osteogenic potential to promote hard tissue formation. In this study, APC-CT was studied as a newly developed material designed for endodontic applications, and therefore, its biological interaction with SHED was investigated. SHED are dental MSCs involved in tooth growth and repair. SHED are easily accessible sources of stem cells offering a valuable opportunity to evaluate the therapies for dental diseases dependent on stem cells due to their clinical relevance [26,31]. 

Cell interaction with the material is highly determined by the surface topography of the material. A smooth surface such as that of nanostructured substrates favors cell attachment and proliferation [32]. In this study, FESEM observation showed that the material surfaces consisted of grains in various sizes with a coral-like appearance, which was in accordance with APC of a previous study [10] and fast-set PC [29]. SHED proliferated and spread directly on the material surfaces. The cells exhibited flattened morphology with extended processes, showing an effective adaptation to the material and affirming favorable interaction and material biocompatibility. Some CSCs such as APC, MTA and fast-set MTA resulted in similar flattened characteristics in seeded human osteosarcoma cells (SaOS-2), osteoblasts and HPLFs, respectively [10,33,34], to those observed in this study. In addition, CT-incorporated scaffold such as HA/chitosan has been reported to support proliferation and attachment of HPLCs [35]. CT supports cell attachment by the interaction of positively charged CT-incorporated material with negatively charged cells; this electrostatic interaction would also enhance cell attachment by the adsorption of certain proteins [36].

Biocompatibility is a major criterion in the application of endodontic material to avoid the adverse effects of the material on the pulp and periodontal tissues which ultimately affect the healing process by cell necrosis or apoptosis [37,38]. Results of the apoptosis assay demonstrated that the APC and APC-CT materials did not induce early, or late apoptosis compared to control, and the percentage of cell viability was increased on days 2 and 3. Overall, the results indicated that APC and APC-CT are nontoxic and have no adverse effect on SHED proliferation. Cornélio et al. [23] found that PC with or without radiopacifiers induced a necrosis cell death at high concentration (100 mg/mL), whereas no cell apoptosis was induced at lower concentrations (10 mg/mL or 1 mg/mL), which was in accordance with our finding. A previous study by Wiegand et al. [39] showed that CT induced apoptotic cell death that was mediated by activation of the effector caspases 3/7 in human keratinocyte cells. Moreover, Gorduysus et al. [38] revealed that high cell viability was observed in response to MTA, while other endodontic materials such as Diaket, Endion and CYMED 8410 induced high percentages of apoptosis. The dissolution of calcium silicate material is correlated with elevation of surrounding pH due to the formation of calcium hydroxide (Ca(OH)_2_), which separates and releases Ca and OH ions [40]; the alternation of extracellular pH could induce cell death due to the change of the potential across the cell membrane and inhibition of ion exchange. Additionally, a possible inducer of cell apoptosis when using CSCs is excess calcium ions, which promote endoplasmic reticulum stress [41]. 

The potential of the experimental material to induce dentinogenic and osteogenic differentiation of SHED was investigated by assessing the expression of the related gene markers [27,42]. Our results revealed that expression of dentinogenic markers *DSPP, MEPE* and *DMP-1*, which have a role in early odontoblastic differentiation and late dentin mineralization [42], was upregulated in SHED treated with CT-containing materials in long-term culture and was higher than that in APC in APC-0.6%CT and APC-2.5%CT groups, indicating the enhancement effect of CT on odontogenic differentiation. The protonated free amino and hydroxyl groups of CT that are released after dissolving and form ionic complexes with a wide variety of spices may be responsible for the effects [43,44]. PC and MTA have been reported to stimulate the expression of *DSPP* and *DMP-1* in hDPSCs on day 14 [14], and the incorporation of 5% CaCl_2_ into MTA improved *DSPP* expression [45]. Moreover, a previous study [46] concluded that CT and dexamethasone with or without vitamin D3 are synergistic dentinogenic inducers when added into MTA and applied in exposed dog teeth, where CT induced *DSPP* upregulation after 7 and 21 days and *MEPE* increase (with no significant difference) after 7 days, which supports the dentinogenic effects of CT found in this study. 

*OPN* and *OCN* are late-stage markers of osteoblast differentiation [47,48]. *OPN* expression was upregulated in SHED treated with CT-containing materials in long-term culture and was higher in SHED treated with APC-0.6%CT and APC-2.5%CT compared to APC. The results also showed that *OCN* expression was upregulated early on day 3 and was higher in SHED treated with CT-containing materials. The results of both genes highlighted the role of CT in osteogenic induction. It was found that PCs from different origins have different potentials of *OPN* expression in DPSCs, which was enhanced by CaCl_2_.2H_2_O addition [49]. Furthermore, *OCN* upregulation in hDPSCs has been indicated when treated with PC and MTA [14]. Lee et al. [50] reported that 10% calcium chloride is able to enhance *OCN* expression in MC3T3-E1 cells when mixed with MTA due to the calcium release. In addition, Ho et al. [51] demonstrated the potential of CT nanofibers in promoting *OPN* and *OCN* expression in osteoblasts.

*RANKL,* a membrane-bound homotrimeric protein, is involved in osteoclast fusion and differentiation via binding to receptor activator of nuclear factor kappa B (RANK) on osteoclast. *OPG* inhibits the differentiation of osteoclasts by binding to *RANKL* and blocking its association with RANK to protect the bone from excessive absorption. Hence, the ratio of *RANKL* and *OPG* plays a primary role in bone mass and strength [52]. The expression of *OPG* and *RANKL* increased on day 14, and approximately similar expression levels were recorded in all the groups. This finding could be explained by data from Coon et al. [53] which showed similar expression levels of *OPG* and *RANKL* when treated with MTA, suggesting that bone resorption is not stimulated directly, but instead, a delayed periradicular tissue healing could occur. 

The transcription factor *RUNX2* is important in odontoblast and osteoblast differentiation in addition to its role in regulating the expression of various tooth- and bone-related genes. It also directs the mesenchymal stem cells to odontoblast or osteoblast lineage [54,55]. *RUNX2* is expressed in the early stage of odontoblast and osteoblast differentiation and then decreased in late-stage and fully differentiated cells [27,56]. Our findings showed that RUNX2 was upregulated in the early differentiation stage (day 7) and downregulated in the late odontoblast/osteoblast differentiation stage (day 14) to achieve a complete differentiation process because RUNX2 expression at the late stage inhibits odontoblast/osteoblast maturation, but some level of expression is required for the expression of the genes encoding the bone matrix proteins [57,58]. Additionally, at the late stage of cell differentiation, elevated RUNX2 expression inhibited odontoblast terminal differentiation and promoted transdifferentiation into osteoblasts [59]. In this study, upregulation of odontoblast markers (DSPP, MEPE and DMP-1) in addition to OPN and OCN, which indicate late-stage osteoblast differentiation, indicated odontoblast/osteoblast lineage differentiation. Furthermore, during odontoblast differentiation and maturation, RUNX2 expression is reported to decrease while DSPP expression increases [60], corroborating our findings. This finding was consistent with a previous study [61] which showed that VEGF/chitosan/ß-GP hydrogel contributed to odontogenic differentiation of DPSCs and the expression of *RUNX2* was increased after 7 days and then decreased on day 14.

*ALP* and *COL1A1* are markers of early odontoblast and osteoblast differentiation [62,63,64,65]. The results showed that the expression of *ALP* was downregulated in all CT-containing material groups, whereas *COL1A1* expression was downregulated in all experimental groups. An et al. [66] found that *ALP* expression is sensitive to high Ca^2+^ and P ion concentrations and decreases in response to increased extracellular Ca^2+^ and P ions. The results also showed that ALP activity was decreased but *RUNX2* expression, cell viability and mineralized matrix formation were improved, showing no evidence of a linear relationship between expression of *ALP* and formation of extracellular mineralization. In accordance with our results, Zanini et al. [67] induced odontoblast differentiation in immortalized murine pulp cells (OD-21) with Biodentine and found that the *COL1A1* and *ALP* expression levels and ALPase activity were decreased while a statistically increase in biomineralization was shown. Interestingly, CaCl_2_, a component of Biodentine and also our experimental APC-CT, has been found to decrease *ALP* expression in a dose-dependent manner as a result of increased Ca^2+^ ions [68]. 

This study showed that the expression levels of *ALP* and *COL1A1* markers, which are involved in early differentiation and related to the intense secretory activity of cells, were decreased, whereas expression levels of markers related to mature odontoblasts, late differentiation and mineralization were increased, indicating that the cells reached a quiescent phase [67,69] and suggesting cell differentiation toward the odontoblast/osteoblast pathway, indicating that APC-CT induced odontoblast/osteoblast differentiation in SHED.

Crystalline calcium phosphate is involved in the mineralization process alongside the collagen and noncollagenous proteins [70]. The mineral deposition secreted by odontoblast/osteoblast is mainly composed of calcium phosphate in the form of hydroxyapatite [71,72]. In the present study, a marked induction of mineralization was detected in SHED treated with APC and APC-CT as revealed by ARS and von Kossa stains, which confirms the dentinogenic/osteogenic differentiation of SHED. The highest amount of mineralization was found in APC-2.5%CT-treated SHED. This amount was reduced gradually in materials containing lower CT concentrations and showed a minimum amount in APC. The mineralization potential of APC in this study was consistent with previous studies in which PC induced the mineralization activity in HDPCs [73] and PDL cells [74]; both studies demonstrated higher mineralization when incubation time increased. Silva et al. [75] reported that PC (referred to as “calcium silicate cement” in the study) incorporated with microparticulate and nanoparticulate zirconium oxide induced continuous dense Von Kossa-positive structures in vivo when the material was implanted into the dorsal skin of rats for 60 days. Another study by Lee et al. [50] showed that CaCl_2_ incorporation into PC-based material achieved similar mineralization results and enhanced the osteogenic effect in osteoblasts. CaCl_2_ incorporation into PC-based materials was reported to stimulate higher calcium release [76], which clarifies the favorable outcomes of some endodontic materials containing CaCl_2_ [77]. Our data showed that CT addition into the material enhanced greater mineralization formation by SHED. This finding was in agreement with previous studies which revealed a positive effect of CT on mineralization [78,79,80]. 

According to the findings of this study, APC-CT improved the dentinogenic/osteogenic capabilities of SHED. It is suggested that CT can be employed to improve the biomineralization of composite materials because CT promotes calcium/phosphate ion accumulation and improves the biomineralization capability of APC-CT. CT promotes mineralization by upregulating dentinogenic/osteogenic differentiation and mineralization genes. The results indicated that CT significantly increased the expression of DSPP, MEPE, DMP-1, OPN, OCN and OPG, indicating that CT has a favorable impact on the promotion of dentinogenic/osteogenic gene expression. In light of the above findings, we suggest that the release of calcium/phosphate ions and the high pH, in addition to the effect of CT in APC-CT material, influenced the dentinogenic/osteogenic capability and biomineralization in vitro. Although our findings show a promising impact on dentinogenic/osteogenic differentiation properties, several limitations need to be addressed. The effects of APC-CT on signaling pathways and upstream and downstream regulatory proteins remain unknown. The current study is also limited to the evaluation of the biological properties of APC-CT in vitro. Further studies are required to explore the clinical application potential of the material in vivo and validate the findings presented in this study.

## 5. Conclusions

The experimental APC-CT material exhibited satisfactory biocompatibility with SHED, maintaining favorable cell viability and attachment, and did not induce apoptotic cell death. Moreover, APC-CT promoted dentinogenic/osteogenic capability in SHED via upregulating the expression levels of dentinogenic and selected osteogenic markers and induced high extracellular matrix mineralization, suggesting that APC-CT might be effective in endodontic applications. Further studies are needed to evaluate the outcomes and effectiveness of this material in clinical applications. 

## Figures and Tables

**Table 1:** Primer sequences and annealing temperatures of individual genes used in qRT-PCR.

Genes	Gene Full Name	Primer Sequences 5′ to 3′	Annealing Temperature (°C)	Accession Number
*ALP*	Alkaline phosphatase	F: GACCTCCTCGGAAGACACTC R: TGAAGGGCTTCTTGTCTGTG	57.4	NM_00478.5
*COL1A1*	Collagen type 1 α 1	F: ACATGTTCAGCTTTGTGGACC R: TGATTGGTGGGATGTCTTCGT	55.6	NM_000088.3
*RUNX2*	Runt-related transcription factor 2	F: TCTTAGAACAAATTCTGCCCTTT R: TGCTTTGGTCTTGAAATCACA	52.4	NM_001024630.3
*OPN*	Osteopontin	F: CAGCCATGAATTTCACAGCC R: GGGAGTTTCCATGAAGCCAC	60.0	NM_000582
*OCN*	Osteocalcin	F: CTCACACTCCTCGCCCTATT R: GTCAGCCAACTCGTCACAGT	60.4	NM_199173
*OPG*	Osteoprotegerin	F: CCACTACGACCTACTGGATGC R: GTTGCCGAAGTCACAGGTG	60.4	NM_002546.3
*RANKL*	Receptor activator of nuclear factor kappa-B ligand	F: GGGTGGAGGTGTACTATGATGG R: CTTGCCGTAGGAGGAGCTG	60.4	NM_033012.3
*GAPDH*	Glyceraldehyde-3-phosphate dehydrogenase	F: CATCATCCCTGCCTCTACTG R: GCCTGCTTCACCACCTTC	57.4	NM_002046
*β-actin*	*β -* actin	F: TCCCTGGAGAAGAGCTACG R: GTAGTTTCGTGGATGCCACA	60.0	NM_001101.3
*MEPE*	Matrix extracellular phosphoglycoprotein	F: CCCTGGAAGAGAAGGAAACAGA R: TGAAACTCAACCTTCCCTTGGT	60.0	NM_001184694.2
*DSPP*	Dentin sialophosphoprotein	F: GGGATGTTGGCGATGCA R: CCAGCTACTTGAGGTCCATCTTC	60.0	NM_014208.3
*DMP-1*	Dentin matrix protein 1	F: TGCAGCTGAGATAGTTCCTAA R: TGTAGCTTTGTGGGTCCTT	60.0	NM_001079911.2

## Data Availability

All data are available within the article.
